# Correlation of Thoracic HRCT Scores with Right Heart Mechanics and TAPSE/sPAP Ratio in Pulmonary Alveolar Proteinosis

**DOI:** 10.3390/jcm15135150

**Published:** 2026-07-02

**Authors:** Omer Ozkan Duman, Lale Duman

**Affiliations:** 1 Department of Cardiology, Izmir Demokrasi University, Buca Seyfi Demirsoy Training and Research Hospital, Izmir 35390, Turkey; 2 Department of Radiology, Dr. Suat Seren Chest Diseases and Surgery Training and Research Hospital, Izmir 35170, Turkey; laleemin@gmail.com

**Keywords:** pulmonary alveolar proteinosis, RV-PA coupling, TAPSE/sPAP, high-resolution computed tomography, right ventricular dysfunction

## Abstract

**Background:** Pulmonary Alveolar Proteinosis (PAP) is a rare interstitial lung disease characterized by intra-alveolar accumulation of lipoproteinaceous material. Although its radiologic features are well documented, the disease’s impact on cardiovascular mechanics—specifically right ventricular (RV) adaptation—remains underexplored. This study aims to explore the correlation between radiologic severity of parenchymal involvement, quantified by high-resolution computed tomography (HRCT) scores, and right heart hemodynamics, with a focus on RV-pulmonary artery (RV-PA) coupling and interventricular interaction. **Methods:** This retrospective observational study analyzed 13 adult patients with confirmed PAP and 70 age- and sex-matched healthy controls. All participants underwent concurrent thoracic HRCT and transthoracic echocardiography. Structural lung damage and “crazy-paving” patterns were quantified using a total lobar HRCT score ranging from 0 to 30. Echocardiographic evaluation assessed right heart hemodynamics, using the tricuspid annular plane systolic excursion-to-systolic pulmonary artery pressure ratio (TAPSE/sPAP) as an index of RV-PA coupling and the RV/LV ratio. Given the small sample size, *p*-values for correlations were adjusted for multiple comparisons using the Bonferroni method, and findings should be regarded as exploratory. **Results:** Left ventricular parameters were preserved across both groups. However, PAP patients had significantly impaired right heart indices compared with controls, including a larger RV basal diameter (37.2 ± 2.3 vs. 32.6 ± 1.6 mm, *p* < 0.001), higher sPAP (38.5 ± 3.5 vs. 23.6 ± 2.9 mmHg, *p* < 0.001), and a higher RV/LV ratio (0.98 ± 0.19 vs. 0.60 ± 0.06, *p* < 0.001). Furthermore, the RV-PA coupling marker, TAPSE/sPAP, was markedly reduced in the PAP cohort (0.43 ± 0.07 vs. 0.88 ± 0.13 mm/mmHg, *p* < 0.001). After Bonferroni correction, elevated HRCT scores remained strongly associated with a higher RV/LV ratio (r = 0.89, *p* < 0.001) and a lower TAPSE/sPAP ratio (r = −0.90, *p* < 0.001). Subgroup analysis indicated that patients with severe radiological scores had more pronounced RV enlargement and lower RV-PA coupling reserves. **Conclusions:** In this exploratory analysis, radiographic severity of alveolar lipoproteinaceous accumulation in PAP was strongly associated with isolated right ventricular dysfunction and impaired RV-PA coupling, despite preserved left heart function. These hypothesis-generating findings suggest that echocardiographic assessment of TAPSE/sPAP and RV/LV ratios may be useful for the clinical follow-up of PAP patients; however, causality cannot be inferred from this small, retrospective, single-center cohort, and larger prospective studies are warranted.

## 1. Introduction

Pulmonary Alveolar Proteinosis (PAP) is a rare interstitial lung disease characterized by intra-alveolar accumulation of lipoproteinaceous material, resulting from disruptions in the granulocyte-macrophage colony-stimulating factor (GM-CSF) signaling pathway, alveolar macrophage dysfunction, and impaired surfactant clearance [1,2]. Although PAP can be classified into autoimmune, secondary, and congenital forms, the vast majority of cases are autoimmune. For diagnosis and assessment of radiological disease severity, high-resolution computed tomography (HRCT)—which typically demonstrates the characteristic “crazy-paving” pattern of ground-glass opacities (GGO) superimposed on thickened interlobular septa—is considered the gold standard [3]. The radiological appearance may also provide clues to etiology; secondary PAP usually exhibits more diffuse involvement, whereas the autoimmune form tends to show a patchier distribution. Recently, researchers have used automated software to accurately quantify the volume of affected lung tissue. These quantitative CT severity scores have shown strong correlation with pulmonary function tests, particularly diffusing capacity for carbon monoxide (DLCO) and forced expiratory volume in one second (FEV1) [4,5]. Clinically, the dense pulmonary infiltrates in PAP can mimic cardiogenic pulmonary edema; however, PAP does not cause cardiomegaly or pleural effusion. Nevertheless, the massive intra-alveolar accumulation of cholesterol raises important questions regarding cardiovascular loading. While the radiological course and parenchymal involvement patterns of PAP have been extensively described in the literature using quantitative CT severity scores, the impact and correlation of these scores with the cardiovascular system and myocardial mechanics have not been adequately addressed.

From a cardiologic and pathophysiological perspective, progressive parenchymal damage, chronic hypoxemia, and decreased intrapulmonary blood flow secondary to PAP insidiously increase pulmonary vascular resistance (PVR), ultimately predisposing to the development of Group 3 Pulmonary Hypertension (PH) [6,7,8]. This progressive afterload increase, triggered by chronic hypoxic vasoconstriction and vascular remodeling, imposes substantial hemodynamic stress on the thin-walled right ventricle (RV) [9]. Although left ventricular ejection fraction (LVEF) and left heart volumetric indices may appear within normal limits during the early stages of the disease, the adaptive myocardial response of the right heart to increased systolic pulmonary artery pressure (sPAP) is the principal determinant of patients’ exercise capacity, morbidity, and clinical prognosis [10].

Echocardiography plays a pivotal role in the noninvasive assessment of the right ventricle’s (RV) mechanical adaptation—known as RV-pulmonary artery (RV-PA) coupling—to increased afterload [11]. Among primary indicators of right ventricular longitudinal systolic function, the ratio of tricuspid annular plane systolic excursion (TAPSE) to sPAP (TAPSE/sPAP) is a highly sensitive marker that reflects the mechanical interplay between right heart contractility and pulmonary vascular load [12,13]. In the literature, impairment of the TAPSE/sPAP ratio has been shown to be a robust, independent, and early predictor of clinical deterioration and mortality in conditions with increased RV load, including pulmonary arterial hypertension [14], acute pulmonary embolism [15], and systemic sclerosis [16,17].

Although the prognostic impact of RV-PA coupling impairment in chronic lung diseases is well recognized, there is a lack of clinical studies specifically examining the direct quantitative relationship between thoracic HRCT scores—reflecting the severity of radiological parenchymal involvement—and comprehensive right heart hemodynamics and RV-PA coupling in patients with PAP [15,16]. Therefore, the aim of this study is to investigate the correlation between the radiological extent of alveolar lipoproteinaceous accumulation (HRCT scores) and echocardiographic parameters reflecting right ventricular performance, RV-PA coupling (TAPSE/sPAP), and interventricular interaction (RV/LV ratio), thereby clarifying the effects of parenchymal obliteration on cardiac mechanics in patients with PAP [16,17].

## 2. Materials and Methods

### 2.1. Study Design and Ethical Approval

This retrospective observational study analyzed medical records of patients diagnosed with PAP who were followed at a tertiary care center between 2010 and 2025. The study protocol was approved by the Clinical Research Ethics Committee of Izmir Demokrasi University Buca Seyfi Demirsoy Training and Research Hospital (Date: 25 February 2026, Decision No: 2026/02-25). All research procedures involving human subjects were conducted in full accordance with the current ethical principles of the Declaration of Helsinki.

### 2.2. Patient Selection and Grouping

**Figure 1:**
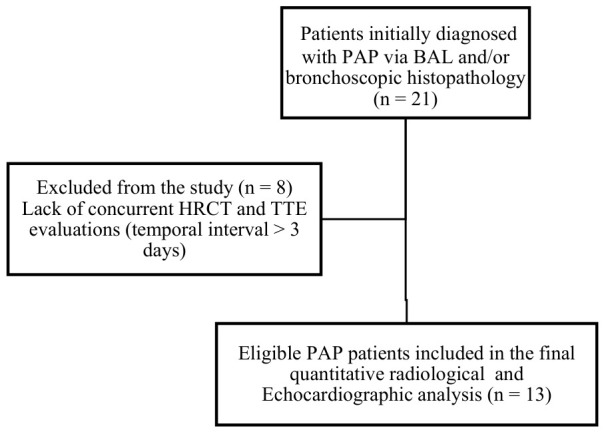
Patient selection flowchart. (PAP = pulmonary alveolar proteinosis, BAL = bronchoalveolar lavage, HRCT = high-resolution computed tomography, TTE = transthoracic echocardiography).

A total of 21 adult patients with a confirmed diagnosis of PAP, established by histopathological analysis of bronchoalveolar lavage (BAL) or lung biopsy, were initially identified. Strict inclusion criteria were applied to ensure physiological validity: only patients who underwent both HRCT and transthoracic echocardiography (TTE) within a 3-day interval were included, minimizing errors from spontaneous disease fluctuations. As a result, 8 patients were excluded, yielding a final cohort of 13 PAP patients (Figure 1). The control group comprised 70 healthy individuals without known cardiopulmonary disease and with high-quality TTE and HRCT images available in the hospital system, matched to the PAP group by age and sex. To ensure reliable assessment of cardiac parameters, individuals with significant valvular disease (moderate-to-severe stenosis or regurgitation), left heart failure (left ventricular ejection fraction < 50%), documented coronary artery disease, chronic arrhythmia (such as atrial fibrillation), or inadequate echocardiographic acoustic windows for quantitative analysis were excluded.

To address the statistical power of this rare-disease cohort, a post hoc sensitivity analysis was performed. With a final sample of 13 PAP patients and 70 controls (α = 0.05, two-tailed, power = 0.80), the design was sufficient to detect between-group standardized effect sizes of Cohen’s d ≈ 0.85 or larger. Because the primary right heart comparisons yielded very large effect sizes (Cohen’s d > 2.5), the achieved power for these endpoints exceeded 0.99. Nevertheless, given the limited sample size, all results should be interpreted as exploratory and hypothesis-generating.

### 2.3. Echocardiographic Evaluation

All transthoracic echocardiographic examinations were performed by specialist cardiologists unaware of clinical data, using a GE Vivid ultrasound system (Vivid 3, GE Systems, Oslo, Norway) equipped with a standard phased array transducer and in accordance with the latest guidelines of the American Society of Echocardiography (ASE) and the European Association of Cardiovascular Imaging (EACVI) [18,19]. LVEF was calculated using the modified Simpson biplane method from apical four- and two-chamber views. Left atrial (LA) diameter, RV basal diameter, and left ventricular volume indices (LVEDV index, LVESV index) were indexed to body surface area (BSA) and reported in mL/m^2^. The following specific parameters were used to assess right heart hemodynamics and RV-PA coupling: systolic pulmonary artery pressure (sPAP), calculated using continuous-wave Doppler across the peak tricuspid regurgitation jet with right atrial pressure estimated from inferior vena cava diameter and respiratory variation (simplified Bernoulli equation, 4v^2^ + RAP); TAPSE, measured from the apical four-chamber view with the M-mode cursor aligned with the lateral tricuspid annulus; the TAPSE/sPAP ratio (mm/mmHg) as an indicator of RV-PA coupling; and the RV/LV ratio, the ratio of right to left ventricular basal diameters measured at end-diastole.

### 2.4. Radiological Assessment and Thoracic HRCT Scoring

All thoracic HRCT images were analyzed by an experienced thoracic radiologist who was blinded to clinical and echocardiographic data. All thoracic HRCT scans were acquired at end-inspiration with the patients in a supine position, utilizing a 64-detector row CT scanner (Brilliance 64, Philips Healthcare, Best, The Netherlands). The standard acquisition parameters were set as follows: a tube voltage of (120) kVp, automatic tube current modulation (e.g., (100–250) mAs), and a pitch of (0.9). For spatial reconstruction, the images were reconstructed using a high-spatial-frequency (sharp) algorithm with a slice thickness of (1.0) mm and a reconstruction interval of (1.0) mm.

The lung parenchyma was divided into five lobes (right upper, middle and lower; left upper and lower), and each lobe was quantitatively scored for the extent of structural damage, infiltration, and the characteristic “crazy-paving” pattern of PAP. The sum of the lobar scores yielded the “Total HRCT Score” (range: 0–30) [5]. Quantitative three-dimensional histogram analysis of the images was performed using Philips IntelliSpace Portal (version 9.0) software. Initial lung extraction was achieved using a semi-automated threshold-based segmentation algorithm (−1024 to −200 HU). Subsequently, a manual region of interest (ROI) adjustment was meticulously performed by an experienced thoracic radiologist to exclude the central airways, major hilar vessels, and any non-pulmonary structures. Images were analyzed at a slice thickness of 1.0 mm, and no additional resampling or noise-reduction filters were applied prior to the histogram extraction, ensuring the preservation of the original parenchymal densities. The density distribution was quantified by binning the voxel data into specific predefined Hounsfield Unit (HU) thresholds with a bin width of 10 HU: normal aerated lung (−∞ to −750 HU), ground-glass opacities (−750 to −300 HU), and consolidation (−300 to 49 HU).

### 2.5. Statistical Analysis

Statistical analyses were performed using jamovi (Version 2.6) and IBM SPSS Statistics Version 26.0 (IBM Corp., Armonk, NY, USA). Normality of variable distributions was assessed visually with histograms and analytically with the Shapiro–Wilk test. Continuous variables were reported as mean ± standard deviation (SD) or median (interquartile range, IQR), as appropriate, and categorical variables as frequency and percentage (%). Comparisons of continuous variables between PAP and healthy controls were performed using the independent-samples *t*-test, with Welch’s correction applied when Levene’s test indicated unequal variances; Cohen’s d was reported as the effect size. Categorical variables were compared using the χ^2^ test of association or Fisher’s exact test when expected cell counts were small. The direction and strength of associations between thoracic CT scores and echocardiographic parameters were assessed using Pearson correlation coefficients. To account for the multiplicity of comparisons in this small cohort, correlation *p*-values were adjusted using the Bonferroni method. A two-sided *p*-value < 0.05 was considered statistically significant. Given the limited sample size (n = 13) of this rare-disease cohort, predictive threshold analyses, such as ROC curves, were deliberately omitted to avoid overfitting and complete separation bias, and all findings are interpreted as exploratory.

## 3. Results

### 3.1. Demographic and Baseline Clinical Characteristics

The study included 13 PAP patients who fully met the inclusion and exclusion criteria, as well as 70 healthy controls matched for age and sex. There were no statistically significant differences between the groups in age (42.69 ± 8.88 vs. 42.59 ± 5.79 years, *p* = 0.956), sex distribution (male: 61.5% vs. 62.9%, χ^2^(1) = 0.01, *p* = 0.928; Fisher’s exact *p* = 1.000), body mass index (26.39 ± 2.96 vs. 26.59 ± 2.66 kg/m^2^, *p* = 0.806), or smoking rates (61.5% vs. 61.4%, χ^2^(1) < 0.01, *p* = 0.994). The basic demographic characteristics of the study population are summarized in Table 1.

### 3.2. Comparison of Left and Right Heart Echocardiographic Parameters

Comprehensive echocardiographic results for both groups are shown in Table 2. There were no significant differences between the PAP and control groups in LVEF, left atrial (LA) diameter, or left ventricular volume indices (LVEDV, LVESV) (all *p* > 0.05). In contrast, parameters reflecting right heart structure and function were significantly impaired in the PAP group. Compared with controls, PAP patients had significantly larger RV basal diameter (37.15 ± 2.30 vs. 32.63 ± 1.64 mm, *t*(81) = 8.53, *p* < 0.001, d = 2.58) and higher sPAP (38.46 ± 3.53 vs. 23.60 ± 2.86 mmHg, *t*(81) = 16.60, *p* < 0.001, d = 5.01). TAPSE was markedly reduced in PAP (16.39 ± 1.33 vs. 20.49 ± 1.67 mm, *p* < 0.001, d = 2.52), and the TAPSE/sPAP ratio was dramatically lower in PAP patients (0.43 ± 0.07 vs. 0.88 ± 0.13 mm/mmHg, *p* < 0.001, d = 4.30). The RV/LV ratio was also significantly higher in PAP (0.98 ± 0.19 vs. 0.60 ± 0.06, *p* < 0.001, d = 2.66). All right heart effect sizes were very large (Cohen’s d > 2.5), indicating robust between-group separation despite the limited sample size.

### 3.3. Quantitative CT and Radiological Findings

**Figure 2:**
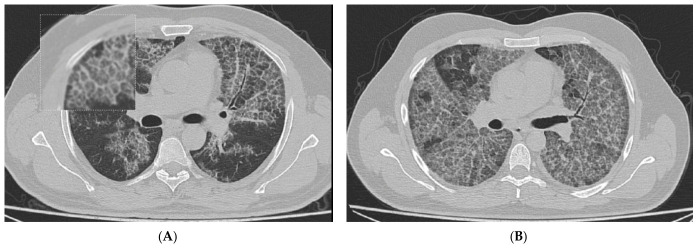
Representative thoracic HRCT images of a PAP patient from the study cohort. (**A**,**B**) Axial sections show extensive, bilateral ground-glass opacities superimposed on a network of thickened septa. This “crazy-paving” pattern visually represents the heavy intra-alveolar lipoproteinaceous accumulation measured in our quantitative analysis.

Automated three-dimensional histogram analysis showed that patients with PAP had significantly reduced aerated lung volume. Most individuals in the control group had normal lung tissue attenuation values (−∞ to −750 HU), whereas PAP patients had substantially greater lung volumes in the ground-glass opacity (GGO) range (−750 to −300 HU) and the consolidation range (−300 to 49 HU) (*p* < 0.001). All 13 PAP patients exhibited widespread, bilateral GGO with septal thickening, consistent with the classic “crazy-paving” pattern (Figure 2). The mean total thoracic HRCT score, reflecting the extent of lung involvement, was 18.5 ± 6.0 in the PAP group.

### 3.4. Correlation Between Thoracic HRCT Scores and Echocardiographic Parameters

**Figure 3:**
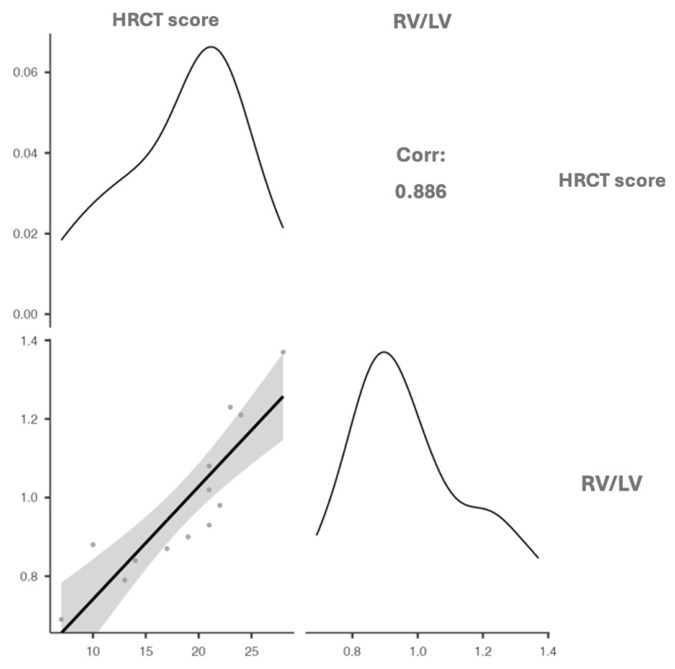
Scatter plot demonstrating the significant positive correlation (r = 0.886, *p* < 0.001) between the HRCT total score and the RV/LV ratio. The linear regression fit (black line) highlights that advanced alveolar lipoproteinosis and higher radiological scores are directly linked to right ventricular dilatation relative to the left ventricle. HRCT = High-resolution computed tomography; RV = Right ventricle; LV = Left ventricle.

**Figure 4:**
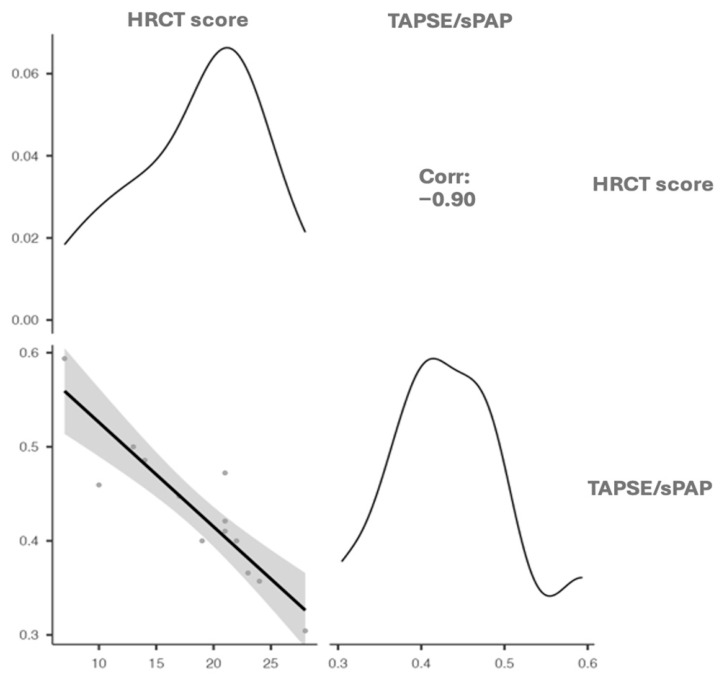
Scatter plot illustrating the strong negative correlation (r = −0.90, *p* < 0.001) between the HRCT total score and the TAPSE/sPAP ratio in patients with Pulmonary Alveolar Proteinosis. The black line represents the linear regression fit, demonstrating that an increase in radiological parenchymal involvement severity is significantly associated with impaired right ventricular-pulmonary arterial (RV-PA) coupling. HRCT = High-resolution computed tomography; TAPSE = Tricuspid annular plane systolic excursion; sPAP = Systolic pulmonary artery pressure.

Correlation analysis was performed within the PAP cohort (n = 13). No correlation was observed between the thoracic HRCT score and left heart parameters (LVEF r = −0.21, *p* = 0.500; LA r = 0.29, *p* = 0.328). In contrast, a strong association was observed between right heart function and the extent of lung involvement on imaging. After Bonferroni correction for seven comparisons (adjusted α = 0.0071), increases in the HRCT total score remained significantly correlated with higher sPAP (r = 0.87, *p* < 0.001) and RV/LV ratio (r = 0.89, *p* < 0.001) (Figure 3), and with lower TAPSE (r = −0.91, *p* < 0.001) and TAPSE/sPAP (r = −0.90, *p* < 0.001) (Figure 4). The correlation with RV basal diameter (r = 0.69, uncorrected *p* = 0.009) did not survive Bonferroni correction (corrected *p* = 0.063). These findings indicate that greater severity of alveolar involvement is associated with impaired right ventricular adaptation. Table 3 summarizes the complete correlation analysis.

### 3.5. Severity Subgroups

To assess clinical implications, we divided PAP patients into two subgroups based on the median HRCT score: a mild-to-moderate group (n = 6) and a severe group (n = 7). Age, gender, and smoking history were comparable between the two cohorts. The overall symptom distribution did not differ significantly between subgroups (χ^2^(2) = 6.10, *p* = 0.047; Fisher’s exact *p* = 0.062). Their echocardiographic profiles, however, differed markedly (Table 4).

Compared with the mild-to-moderate group, the severe group had significantly higher sPAP (40.29 ± 3.20 vs. 36.33 ± 2.73 mmHg, *p* = 0.037, d = 1.32), lower TAPSE (*p* = 0.009, d = 1.76), a substantially lower RV-PA coupling index (TAPSE/sPAP 0.39 ± 0.05 vs. 0.48 ± 0.07, *p* = 0.018, d = 1.54), and a higher RV/LV ratio (1.12 ± 0.16 vs. 0.83 ± 0.08, *p* = 0.002, d = 2.26). RV basal diameter showed a borderline difference (*p* = 0.050). These results underscore that extensive alveolar filling is associated with greater right heart dysfunction.

Individual-level demographic, radiological, and echocardiographic data for all 13 PAP patients are provided in Supplementary Table S1.

### 3.6. Therapeutic Approaches and Clinical Outcomes

Regarding clinical management, all 13 PAP patients in our cohort underwent whole lung lavage (WLL). The procedure was generally well-tolerated. However, 3 patients with severely reduced baseline TAPSE/sPAP ratios experienced transient perioperative right heart strain requiring temporary inotropic support. Ultimately, all patients demonstrated significant symptomatic improvement post-lavage without severe or irreversible acute cardiovascular decompensation. Due to the retrospective nature of this study, structured long-term follow-up data regarding the patients’ subsequent clinical courses were unavailable.

## 4. Discussion

This study provides further insights into the limited existing literature regarding the relationship between thoracic HRCT scores—reflecting the severity of parenchymal radiological involvement—and right heart mechanics and RV-PA coupling in patients with the rare interstitial lung disease Pulmonary Alveolar Proteinosis. The most striking finding is a strong negative correlation (r = −0.90) between the HRCT score and the TAPSE/sPAP ratio, as well as a strong positive correlation (r = 0.89) with the RV/LV ratio, which persisted after Bonferroni correction for multiple comparisons. Despite extensive lipoproteinaceous accumulation in the lung parenchyma, preservation of left heart function (LVEF, LA, left heart volume indices) indicates that the cardiovascular effects of PAP appear to be primarily determined by right ventricular hemodynamics and myocardial adaptation. Because this analysis is correlational, these associations should not be interpreted as evidence of direct causality. Given that isolated right heart dysfunction is a well-established independent predictor of exercise capacity and survival in other interstitial lung diseases, our findings may be relevant for clinical follow-up [20].

The pathophysiology of PAP involves the filling of alveoli with surfactant-like lipoproteinaceous material, leading to widespread ventilation-perfusion mismatch and consequently chronic alveolar hypoxia. This chronic hypoxia triggers hypoxic pulmonary vasoconstriction, causing an insidious increase in pulmonary vascular resistance (PVR) and, over time, vascular remodeling, paving the way for the development of Group 3 Pulmonary Hypertension (PH) [21]. The strong positive correlation observed between HRCT score and sPAP in our study indicates that radiological parenchymal destruction is closely associated with the pulmonary vascular bed and afterload. Similarly, in the pathophysiology of Group 3 PH, the increase in PVR and myocardial stress induced by alveolar hypoxia is well described in the literature and is directly related to right ventricular hypertrophy and loss of functional capacity [6,8,10].

The earliest and most sensitive noninvasive indicator of right ventricular myocardial contractile adaptation (coupling) to increased afterload is the TAPSE/sPAP ratio. In healthy individuals, this ratio is >0.8 mm/mmHg, whereas in our study it decreased to 0.43 ± 0.07 mm/mmHg in PAP patients. Previous studies have shown that a reduced TAPSE/sPAP ratio in conditions such as pulmonary hypertension and systemic sclerosis predicts clinical worsening, elevated NT-proBNP levels, and high mortality long before overt right heart failure develops [16,17]. The low RV-PA coupling ratio observed in our PAP patients suggests that, even if they appear clinically stable at rest, their right ventricular myocardial reserves may be reduced, which could place them at risk of decompensation under exertion or anesthesia. However, the hemodynamic interpretation of the TAPSE/sPAP ratio requires careful consideration of concurrent valvular pathologies, particularly tricuspid regurgitation (TR). In a pressure-overloaded RV, the development of significant secondary TR imposes a superimposed volume overload, which can lead to an exaggerated, pseudo-normal TAPSE value. Concurrently, severe TR may induce an underestimation of sPAP due to the early equalization of right ventricular and right atrial pressures. Consequently, a relevant TR can erroneously inflate the TAPSE/sPAP ratio, potentially misleading prognostic evaluations and clinical decision-making. Although patients with moderate-to-severe valvular regurgitation were strictly excluded from our study, and the profoundly impaired TAPSE/sPAP ratios in our cohort do not suggest the presence of highly relevant confounding TR, this pathophysiological interplay must be carefully considered when evaluating RV-PA coupling in daily clinical practice.

Another finding in our study is that the RV/LV ratio, which was 0.60 in the control group, reached 0.98 ± 0.19 in the PAP group. Pressure and volume overload of the right ventricle lead to right ventricular dilatation and push the interventricular septum toward the left ventricle (interventricular shift). This “interventricular dependence” may restrict left ventricular diastolic filling, thereby reducing overall cardiac output. The main and most effective treatment for PAP, Whole Lung Lavage (WLL), is a hemodynamically challenging procedure requiring general anesthesia and one-lung ventilation [22]. During WLL, the hydrostatic pressure generated by the lavage fluid could theoretically exacerbate impaired RV-PA coupling and the elevated RV/LV ratio. In this regard, our study suggests that, in addition to pulmonary function tests and HRCT findings, quantitative echocardiographic parameters such as TAPSE/sPAP and the RV/LV ratio may be worth evaluating when considering WLL. Whether early WLL intervention in PAP patients with a declining TAPSE/sPAP ratio improves outcomes remains to be established in prospective studies [23].

Our study has several limitations. The retrospective observational design and small sample size (n = 13) are inherent limitations, and the findings should therefore be regarded as exploratory and hypothesis-generating rather than confirmatory. However, PAP is classified as an “ultra-rare” disease, with an estimated prevalence of approximately 6.87 per million [24,25]. A formal a priori sample-size calculation was not feasible given the disease rarity; however, a post hoc analysis confirmed that the very large observed effect sizes (Cohen’s d > 2.5 for the principal right heart endpoints) yielded statistical power exceeding 0.99 for these comparisons. Due to the retrospective design of the study, our cardiac evaluation is based on validated, structured clinical echocardiography reports archived in the hospital system. Raw digital image recordings and video loops were not available for subsequent analysis; consequently, formal within-observer and between-observer variability assessments and the inclusion of representative echocardiographic images could not be performed. Another limitation is that right heart catheterization (RHC), the gold standard for assessing pulmonary hemodynamics, was not routinely performed, and the data relied on TTE-based sPAP measurements; because of the retrospective design, RHC data were not available for any patient. However, previous studies have demonstrated excellent agreement between echocardiographic measurement of the TAPSE/sPAP ratio and invasive RHC data [12]. In addition, owing to the retrospective nature of the study, pulmonary function test results, arterial oxygenation indices (e.g., PaO_2_/FiO_2_), serum anti-GM-CSF antibody titers, and detailed treatment outcomes following whole lung lavage could not be reliably retrieved for the full cohort; the absence of these variables limits assessment of disease-severity scores such as the 2024 ERS Disease Severity Score and represents an important limitation to be addressed in future prospective work. Furthermore, the small number of patients precluded the determination of a definitive clinical cut-off value for the TAPSE/sPAP ratio via ROC analysis. Finally, multiplicity remains a concern in a cohort of this size; although Bonferroni correction was applied to the primary correlation analysis, residual risk of type I error cannot be fully excluded. These limitations should be addressed in future large-scale, multicenter prospective studies.

## 5. Conclusions

In this exploratory, retrospective, single-center study of patients with Pulmonary Alveolar Proteinosis, thoracic HRCT score was strongly associated with both right ventricular dysfunction and impaired RV-PA coupling, despite preserved left-heart function. As radiologic parenchymal involvement became more extensive, the TAPSE/sPAP ratio decreased and the RV/LV ratio increased, and these associations remained significant after correction for multiple comparisons. Because of the correlational design and small sample size, causality cannot be inferred. These hypothesis-generating findings suggest that incorporating echocardiographic right heart and coupling analyses into the clinical follow-up of PAP patients—and potentially into the timing of invasive treatments such as WLL—may be of value, but this should be confirmed in larger prospective studies.

## Figures and Tables

**Table 1:** Comparison of demographic characteristics between the PAP and control groups.

*Variable*	Control (n = 70)	PAP (n = 13)	Test	*p*
	M ± SD/n (%)	M ± SD/n (%)		
Age (years)	42.59 ± 5.79	42.69 ± 8.88	*t* = 0.06	0.956
BMI (kg/m^2^)	26.59 ± 2.66	26.39 ± 2.96	*t* = 0.25	0.806
Gender, male, n (%)	44 (62.9)	8 (61.5)	χ^2^ = 0.01	0.928
Smoking, yes, n (%)	43 (61.4)	8 (61.5)	χ^2^ < 0.01	0.994

*Note.* Continuous variables are mean ± SD (independent-samples *t*-test); categorical variables are n (%) (χ^2^ test). Fisher’s exact test confirmed all categorical comparisons (Gender *p* = 1.000; Smoking *p* = 1.000). PAP = pulmonary alveolar proteinosis; BMI = body mass index.

**Table 2:** Comparison of echocardiographic parameters between groups.

*Variable*	Control (n = 70)		PAP (n = 13)		*t*	*p*	Cohen’s *d*
	*M*	*SD*	*M*	*SD*			
Age (years)	42.59	5.79	42.69	8.88	0.06	0.956	0.02
BMI (kg/m^2^)	26.59	2.66	26.39	2.96	0.25	0.806	0.07
LVEF (%)	60.71	1.12	60.92	1.26	0.61	0.546	0.18
LA (mm)	33.51	1.62	33.62	2.33	0.19	0.848	0.06
LVEDV index (mL/m^2^)	50.39	4.16	50.31	3.52	0.06	0.950	0.02
LVESV index (mL/m^2^)	23.44	1.66	23.46	1.85	0.04	0.971	0.01
RV (mm)	32.63	1.64	37.15	2.30	8.53	<0.001	2.58
*s*PAP (mmHg)	23.60	2.86	38.46	3.53	16.60	<0.001	5.01
TAPSE (mm)	20.49	1.67	16.39	1.33	8.34	<0.001	2.52
TAPSE/*s*PAP	0.88	0.13	0.43	0.07	17.59	<0.001	4.30
RV/LV ratio	0.60	0.06	0.98	0.19	7.03	<0.001	2.66

*Note.* Values are mean and SD. Student’s *t* test was used for all comparisons except TAPSE/sPAP and RV/LV, for which Welch’s *t* test was reported because Levene’s test was significant. Cohen’s d effect sizes are reported for all variables. LVEF = left ventricular ejection fraction; LA = left atrium; RV = right ventricle; sPAP = systolic pulmonary artery pressure; TAPSE = tricuspid annular plane systolic excursion; BMI = Body mass index; LVEDV = Left ventricular end-diastolic volume; LVESV = Left ventricular end-systolic volume.

**Table 3:** Correlation analysis of HRCT total score with echocardiographic parameters in PAP patients.

*Parameter*	Pearson’s *r*	*p* (Uncorrected)	*p* (Bonferroni)
LVEF (%)	−0.21	0.500	1.000
LA (mm)	0.29	0.328	1.000
RV (mm)	0.69	0.009	0.063
*s*PAP (mmHg)	0.87	<0.001	<0.001
TAPSE (mm)	−0.91	<0.001	<0.001
TAPSE/*s*PAP (mm/mmHg)	−0.90	<0.001	<0.001
RV/LV ratio	0.89	<0.001	<0.001

*Note.* Pearson correlation coefficients (N = 13, df = 11). Bonferroni-corrected *p*-values account for seven simultaneous comparisons (adjusted α = 0.0071); significant associations after correction are shown in bold. The strong negative correlation of the TAPSE/sPAP ratio (r = −0.90) indicates impaired right ventricular-pulmonary arterial coupling, whereas the strong positive correlation of the RV/LV ratio (r = 0.89) highlights right ventricular dilatation. HRCT = high-resolution computed tomography; LVEF = left ventricular ejection fraction; LA = left atrium; RV = right ventricle; sPAP = systolic pulmonary artery pressure; TAPSE = tricuspid annular plane systolic excursion.

**Table 4:** Clinical, radiological, and echocardiographic characteristics of PAP patients, stratified by HRCT severity.

*Variable*	Mild–Mod (n = 6)	Severe (n = 7)	*t*/χ^2^	*p*	Cohen’s *d*
	*M ± SD*/n (%)	*M ± SD*/n (%)			
**Demographics**					
Age (years)	44.83 ± 7.00	40.86 ± 10.42	0.79	0.445	0.44
Gender, female	3 (50.0)	2 (28.6)	0.63	0.592	—
BMI (kg/m^2^)	26.17 ± 3.87	26.57 ± 2.23	0.24	0.818	0.13
Smoking, yes	3 (50.0)	5 (71.4)	0.63	0.592	—
**Clinical symptoms**					
Asymptomatic	2 (33.3)	0 (0.0)	—	0.192	—
Dyspnea	4 (66.7)	3 (42.9)	—	0.592	—
Cough	0 (0.0)	4 (57.1)	—	0.070	—
Overall distribution	—	—	6.10	0.062	—
**Radiological**					
Total HRCT score	13.33 ± 4.41	22.86 ± 2.54	4.87	<0.001	2.71
**Echocardiographic**					
LVEF (%)	61.00 ± 1.26	60.86 ± 1.35	0.20	0.848	0.11
LA (mm)	33.83 ± 2.48	33.43 ± 2.37	0.30	0.770	0.17
RV (mm)	35.83 ± 1.60	38.29 ± 2.29	2.20	0.050	1.22
*s*PAP (mmHg)	36.33 ± 2.73	40.29 ± 3.20	2.37	0.037	1.32
TAPSE (mm)	17.33 ± 1.03	15.57 ± 0.98	3.16	0.009	1.76
TAPSE/*s*PAP	0.48 ± 0.07	0.39 ± 0.05	2.76	0.018	1.54
RV/LV ratio	0.83 ± 0.08	1.12 ± 0.16	4.06	0.002	2.26

*Note.* Continuous variables are mean ± SD (Student’s *t* test, df = 11); categorical variables are n (%). Fisher’s exact test was used for categorical comparisons; the overall symptom distribution yielded χ^2^(2) = 6.10, *p* = 0.047, with Fisher’s exact *p* = 0.062 reported as the primary result. HRCT = high-resolution computed tomography; LVEF = left ventricular ejection fraction; LA = left atrium; RV = right ventricle; sPAP = systolic pulmonary artery pressure; TAPSE = tricuspid annular plane systolic excursion.

## Data Availability

The original contributions presented in this study are included in the article/supplementary material. Further inquiries can be directed to the corresponding author(s).

## Supplementary Materials

The following supporting information can be downloaded at https://www.mdpi.com/article/10.3390/jcm15135150/s1: Table S1: Individual Demographic, Radiological, and Echocardiographic Data of the 13 PAP Patients.
