# Supplementary material for: Correlation of Thoracic HRCT Scores with Right Heart Mechanics and TAPSE/sPAP Ratio in Pulmonary Alveolar Proteinosis
Source: J Clin Med. 2026 Jul 2;15(13):5150. doi: 10.3390/jcm15135150 (PMC13362687; doi:10.3390/jcm15135150)
Supplement: Supplementary file 1 [file jcm-15-05150-s001.zip › jcm-4375403-supplementary.pdf]

Supplementary Materials

Table S1. *Individual Demographic, Radiological, and Echocardiographic Data of the 13 PAP Patients.*

Patient	*Age*	*Sex*	*Smoking*	*BMI*	*HRCT*	*Severity*	*LVEF*	*RV*	*s*PAP	*TAPSE*	TAPSE/*s*PAP	*RV/LV*	*Symptom*
P1	52	F	Yes	26	7	Mild–Mod	60	34	32	19	0.59	0.69	None
P2	46	M	No	21	10	Mild–Mod	63	36	37	17	0.46	0.88	Dyspnea
P3	36	M	No	27	13	Mild–Mod	60	36	36	18	0.50	0.79	None
P4	52	M	Yes	23	14	Mild–Mod	62	34	35	17	0.49	0.84	Dyspnea
P5	37	F	No	32	17	Mild–Mod	61	38	38	17	0.45	0.87	Dyspnea
P6	46	F	Yes	28	19	Mild–Mod	60	37	40	16	0.40	0.90	Dyspnea
P7	48	F	Yes	26	21	Severe	62	40	36	17	0.47	0.93	Dyspnea
P8	41	M	Yes	27	21	Severe	60	37	38	16	0.42	1.02	Dyspnea
P9	21	F	No	24	21	Severe	61	34	39	16	0.41	1.08	Cough
P10	48	M	Yes	29	22	Severe	60	38	40	16	0.40	0.98	Dyspnea
P11	46	M	No	25	23	Severe	63	39	41	15	0.37	1.23	Cough
P12	33	M	Yes	30	24	Severe	61	41	42	15	0.36	1.21	Cough
P13	49	M	Yes	25	28	Severe	59	39	46	14	0.30	1.37	Cough

Note. Patients are anonymized (P1–P13) and ordered by ascending total HRCT score. Severe cases (HRCT score ≥ 21, the cohort median) are shaded. Units: Age (years); BMI (kg/m^2); HRCT score (0–25); RV (mm); sPAP (mmHg); TAPSE (mm); TAPSE/sPAP (mm/mmHg). M = male; F = female.
